# Supplementary material for: The nutritional status of children living within institutionalized care: a systematic review
Source: PeerJ. 2020 Feb 6;8:e8484. doi: 10.7717/peerj.8484 (PMC7007983; doi:10.7717/peerj.8484)
Supplement: Supplemental Information 1 [file peerj-08-8484-s001.docx]

## Systematic Review and/or Meta-Analysis Rationale

For systematic reviews / meta-analyses, authors are asked to provide the following information:

1. The rationale for conducting the meta-analysis;

Heterogeneity in the type of interventions prevented our ability to conduct a meta-analysis of the study, so a narrative synthesis was used.

1. The contribution that the meta-analysis makes to knowledge in light of previously published related reports, including other meta-analyses and systematic reviews.

There have been no previously conducted systematic reviews or meta-analysis conducted on this population, in light of other previous publications we think this systematic review provides a significant highlight to the need for additional research on this topic.
