# Supplementary material for: The nutritional status of children living within institutionalized care: a systematic review
Source: PeerJ. 2020 Feb 6;8:e8484. doi: 10.7717/peerj.8484 (PMC7007983; doi:10.7717/peerj.8484)
Supplement: Appendix S1 [file peerj-08-8484-s002.docx]

Appendix 1: Database Search Strategy and Acronyms

Database Search Strategy:

Orphanage

- Institutionalized care

- Residential Child Care

- Children's Home

Anthropometry

- Stunting

- Wasting

- Underweight

- BMI

- Anemia

Nutrition

- Malnutrition

- Nutritional Status

**OVID Keyword Search: December 30^th^ 2018 - Jan 6^th^, 2019**

1. Orphanage or institutionalized care or residential child care or children’s home
2. Nutrition or nutritional status (MeSH Term) or malnutrition (MeSH)
3. Anthropometry OR (length for age OR length-for-age OR LFA OR LAZ) OR linear growth OR stunted OR stunting OR malnutrition OR wasting OR wasted OR oedematous malnutrition OR edematous malnutrition OR kwashiorkor OR protein-energy malnutrition OR (SAM OR MAM OR GAM) OR weight-for-length OR weight for length OR WFL OR WLZ OR muac OR mid upper arm circumference OR mid-upper-arm-circumference and (low OR small) OR underweight OR thinness OR (weight-for-age OR weight for age OR WFA OR WAZ) OR Anemia OR Anaemia OR Hemglobin Levels OR BMI

Through Ovid, four electronic databases were searched; PubMed/ Medline 1950’s to present, CINHAL PLUS 1937 to present, Global Health Database 1910 to 2018 Week 51 and Embase Classic+Embase 1947 to 2018 December 31.

Acronyms:

BMI: Body mass index

CC: Community children, children living with their biological families

CDC: Centers for Disease Control (USA)

CLS: Children living on the streets

FBC: Family-based care, orphaned, separated, or abandoned children living in family-based care settings like foster care or kinship care.

GAM: Global acute malnutrition

HAZ/ LFA/ LAZ: Height/length for age z-score

IBC: Institution-based care, children living in institutional care/ residential care facilities.

LBW: Low birth weight

MAM: Moderate acute malnutrition

MUAC: Mid-upper arm circumference

NCHS: National Center for Health Statistics (USA)

SAM: Severe acute malnutrition

WAZ/ WFA: Weight-for-age z-score

WHO: World Health Organization

WHZ/ WFL/ WLZ: Weight for height/length z-score
