# Supplementary material for: The nutritional status of children living within institutionalized care: a systematic review
Source: PeerJ. 2020 Feb 6;8:e8484. doi: 10.7717/peerj.8484 (PMC7007983; doi:10.7717/peerj.8484)
Supplement: Appendix S3 [file peerj-08-8484-s004.docx]

Appendix 3: Studies excluded and reasons.

| Author Year | Author |
| --- | --- |
| Johnson, 2018 | Anthropometric information in a different study |
| Johnson, 2011 | Anthropometric information in a different study |
| The St. Petersburg—USA Orphanage Research Team, 2008 | Anthropometric information in a different study |
| Diamond, 2003 | No breakout of IBC |
| He, 2007 | No breakout of IBC |
| Adotey, 2011 | Non-standard or insufficient Anthropometric measurements/measurements of nutrition status |
| Adotey, 2011 | Non-standard or insufficient Anthropometric measurements/measurements of nutrition status |
| Al-Jobair, 2013 | Non-standard or insufficient Anthropometric measurements/measurements of nutrition status |
| Al-Maweri, 2014 | Non-standard or insufficient Anthropometric measurements/measurements of nutrition status |
| Ankita2014 | Non-standard or insufficient Anthropometric measurements/measurements of nutrition status |
| Archelli, 2014 | Non-standard or insufficient Anthropometric measurements/measurements of nutrition status |
| Arpita, 2014 | Non-standard or insufficient Anthropometric measurements/measurements of nutrition status |
| Aurpibul, 2010 | Non-standard or insufficient Anthropometric measurements/measurements of nutrition status |
| Bailey, 2013 | Non-standard or insufficient Anthropometric measurements/measurements of nutrition status |
| Baptista, 2018 | Non-standard or insufficient Anthropometric measurements/measurements of nutrition status |
| Baron, 2001 | Non-standard or insufficient Anthropometric measurements/measurements of nutrition status |
| Barroso Junior, 2006 | Non-standard or insufficient Anthropometric measurements/measurements of nutrition status |
| Blignaut, 2007 | Non-standard or insufficient Anthropometric measurements/measurements of nutrition status |
| Blignaut, 2007 | Non-standard or insufficient Anthropometric measurements/measurements of nutrition status |
| Boondit, 2014 | Non-standard or insufficient Anthropometric measurements/measurements of nutrition status |
| Boontanom, 2014 | Non-standard or insufficient Anthropometric measurements/measurements of nutrition status |
| Bos, 2009 | Non-standard or insufficient Anthropometric measurements/measurements of nutrition status |
| Carr, 2018 | Non-standard or insufficient Anthropometric measurements/measurements of nutrition status |
| Children's Health Care Collaborative Study Group, 1992 | Non-standard or insufficient Anthropometric measurements/measurements of nutrition status |
| Children's Health Care Collaborative Study Group, 1993 | Non-standard or insufficient Anthropometric measurements/measurements of nutrition status |
| Chizoba, 2014 | Non-standard or insufficient Anthropometric measurements/measurements of nutrition status |
| Culha, 2004 | Non-standard or insufficient Anthropometric measurements/measurements of nutrition status |
| Dixit, 2009 | Non-standard or insufficient Anthropometric measurements/measurements of nutrition status |
| El-Wahab, 2015 | Non-standard or insufficient Anthropometric measurements/measurements of nutrition status |
| Freitas-Fernandes, 2002 | Non-standard or insufficient Anthropometric measurements/measurements of nutrition status |
| Golden, 2002 | Non-standard or insufficient Anthropometric measurements/measurements of nutrition status |
| Hersh, 1991 | Non-standard or insufficient Anthropometric measurements/measurements of nutrition status |
| Hong, 2011 | Non-standard or insufficient Anthropometric measurements/measurements of nutrition status |
| Huq, 2013 | Non-standard or insufficient Anthropometric measurements/measurements of nutrition status |
| Inabo, 2011 | Non-standard or insufficient Anthropometric measurements/measurements of nutrition status |
| Isenbarger, 1998 | Non-standard or insufficient Anthropometric measurements/measurements of nutrition status |
| Jagvir, 1997 | Non-standard or insufficient Anthropometric measurements/measurements of nutrition status |
| Kim, 2003 | Non-standard or insufficient Anthropometric measurements/measurements of nutrition status |
| Kim, 2003 | Non-standard or insufficient Anthropometric measurements/measurements of nutrition status |
| Kubiak, 2015 | Non-standard or insufficient Anthropometric measurements/measurements of nutrition status |
| McCall, 2010 | Non-standard or insufficient Anthropometric measurements/measurements of nutrition status |
| Munoz-Hoyos, 2001 | Non-standard or insufficient Anthropometric measurements/measurements of nutrition status |
| Oh, 2010 | Non-standard or insufficient Anthropometric measurements/measurements of nutrition status |
| Oluboyo, 2017 | Non-standard or insufficient Anthropometric measurements/measurements of nutrition status |
| Onigbinde, 2017 | Non-standard or insufficient Anthropometric measurements/measurements of nutrition status |
| Ozkalp, 2010 | Non-standard or insufficient Anthropometric measurements/measurements of nutrition status |
| Pagornrat, 2009 | Non-standard or insufficient Anthropometric measurements/measurements of nutrition status |
| Pintong, 2014 | Non-standard or insufficient Anthropometric measurements/measurements of nutrition status |
| Pruksachatkunakorn, 2002 | Non-standard or insufficient Anthropometric measurements/measurements of nutrition status |
| Ramsha, 2017 | Non-standard or insufficient Anthropometric measurements/measurements of nutrition status |
| Rebello, 2011 | Non-standard or insufficient Anthropometric measurements/measurements of nutrition status |
| Ruta, 1999 | Non-standard or insufficient Anthropometric measurements/measurements of nutrition status |
| Sharma, 2014 | Non-standard or insufficient Anthropometric measurements/measurements of nutrition status |
| Shrestha, 2010 | Non-standard or insufficient Anthropometric measurements/measurements of nutrition status |
| Solarsh, 1996 | Non-standard or insufficient Anthropometric measurements/measurements of nutrition status |
| Stark, 2017 | Non-standard or insufficient Anthropometric measurements/measurements of nutrition status |
| Supriya, 2015 | Non-standard or insufficient Anthropometric measurements/measurements of nutrition status |
| Tande, 2009 | Non-standard or insufficient Anthropometric measurements/measurements of nutrition status |
| Bischof, 2002 | Population |
| Le Thanh, 2012 | Population |
| Chakraborty, 2004 | Population |
| Abe, 2000 | Study Type |
| Cataldo, 2007 | Study Type |
| Frank, 1996 | Study Type |
| Martin, 1998 | Study Type |
| McCall, 2018 | Study Type |
| Al-Shibani, 2009 | Unable to find full text |
| Beard, 2005 | Unable to find full text |
| Bhuvaneswari, 2017 | Unable to find full text |
| Blignaut, 2007 | Unable to find full text |
| Kannan, 2018 | Unable to find full text |
| Makhlouf,1994 | Unable to find full text |
| Virk, 2012 | Unable to find full text |
